# Managing Urban Stroke Health Expenditures in China: Role of Payment Method and Hospital Level

**DOI:** 10.34172/ijhpm.2022.5117

**Published:** 2022-02-22

**Authors:** Yong Yang, Xiaowei Man, Zhe Yu, Stephen Nicholas, Elizabeth Maitland, Zhengwei Huang, Yong Ma, Xuefeng Shi

**Affiliations:** ^1^Medical Device Regulatory Research and Evaluation Center, West China Hospital, Sichuan University, Chengdu, China.; ^2^School of Management, Beijing University of Chinese Medicine, Beijing, China.; ^3^National Institute of Traditional Chinese Medicine Strategy and Development, Beijing University of Chinese Medicine, Beijing, China.; ^4^Guang’anmen Hospital, China Academy of Chinese Medical Sciences, Beijing, China.; ^5^Australian National Institute of Management and Commerce, Sydney, NSW, Australia.; ^6^Guangdong Institute for International Strategies, Guangdong University of Foreign Studies, Guangzhou, China.; ^7^School of Economics and School of Management, Tianjin Normal University, Tianjin, China.; ^8^Newcastle Business School, University of Newcastle, Callaghan, NSW, Australia.; ^9^University of Liverpool Management School, University of Liverpool, Liverpool, UK.; ^10^China Health Insurance Research Association, Beijing, China.

**Keywords:** Payment Method, Health Insurance, Health Expenditure, Out-of-Pocket Payment, Stroke

## Abstract

**Background:** Stroke is one of the leading public health issues in China and imposes a heavy financial burden on patients and the healthcare system. This study assess which payment method provides the lowest hospital costs for China’s healthcare system and the lowest out-of-pocket (OOP) expense for insured patients.

**Methods:** This is a 4-year cross-sectional study. From the China Health Insurance Research Association (CHIRA) database, a 5% random sample of urban health insurance claims was obtained. Descriptive analysis was conducted and a generalized linear model (GLM) with a gamma distribution and a log link was estimated.

**Results:** For outpatients, capitation payment had the lowest hospital cost (RMB180.9/US$28.8) and lowest OOP expenses (RMB75.6/US$12.0) per patient visit in primary hospitals compared with fee-for-service (FFS) payments. The global budget (GB) displayed the lowest total hospital costs (RMB344.7/US$54.8) in secondary hospitals, and was 27.4% (95% CI=-0.32, -0.29) lower than FFS. FFS had the lowest OOP expenses (RMB123.4/US$19.6 vs. RMB151.8/US$24.1) in secondary and tertiary hospitals. For inpatients, FFS had the lowest total hospital costs (RMB5918.7/US$941.1) per visit and capitation payments had the lowest OOP expenses (RMB876.5/US$139.4, 40.1% lower than FFS, 95% CI=-0.58, -0.15) in primary hospitals. Capitation payment had both the lowest hospital costs (RMB7342.9/US$1167.5 vs. RMB17 711.7/US$2816.2) and the lowest OOP expenses (RMB1664.2/US$264.6 vs. RMB3276.3/US$520.9) for both secondary and tertiary hospitals.

**Conclusion:** For outpatients in primary hospitals and inpatients in secondary and tertiary hospitals, the capitation payment was the most money-saving payment method delivering both the lowest OOP expenses for patients and the lowest hospital total costs for hospitals. We recommend that health policymakers prioritize the implementation of the payment method with the lowest OOP expenses when the payment method does not deliver both the lowest hospital costs for the health system and lowest OOP expenses for patients.

## Background

**Key Messages**

** Implications for policy makers**

- For the health system and patient out-of-pocket (OOP) expenses, the capitation payment performed as the superior payment method from the perspective of expenditure, offering both the lowest total hospital costs and the lowest OOP expenses for outpatients in primary hospitals and inpatients in both secondary and tertiary hospitals.
- When the lowest OOP expenses and lowest total hospital costs do not coincide, there was no mutual “best payment method” for the hospital system and patients. Taking the Chinese government’s Healthy China 2030 Outline goal of gradually reducing the proportion of OOP expenses for patients as the primary aim of the payment method, the payment method with the lowest OOP expenses should be adopted.
- For different hospital levels, and for outpatients and inpatients, mixed payment methods should be adopted to reduce OOP expenses for patients and the total hospital costs for the health system.

** Implications for the public**

This study estimated out-of-pocket (OOP) expenses for urban patients with stroke and total hospital costs for the health system under different payment methods and different levels of hospitals in China, to provide recommendations for applying suitable payment methods for hospitals, reduce the financial burden for patients with stroke and to control excessively increasing health expenditures. Additionally, the varied OOP expenses and hospital costs for patients with stroke between different payment methods indicate that the payment methods may shape whether or how much the health services were provided. In particular, different types of health services, which could determine the share of total hospital costs paid by patients. In most cases, although the global budget (GB) had fewer total hospital costs than the fee-for-service (FFS), it incurred higher OOP expenses for patients with stroke.

Stroke is a leading cause of death and years of life lost in China,^1,2^ imposing a heavy financial burden on stroke patients and the health system.^3-5^ Catastrophic out-of-pocket (OOP) medical expense payments exceeding 40 % of a household’s yearly income,^6^ accounted for 17% of all stroke OOP payments, which was higher than the 13% for the overall incidence of catastrophic health expenditure for all illnesses.^7,8^ Catastrophic OOP expenses and stroke health expenditures pushed families into economic distress and poverty. In response to widespread public discontent with prohibitive medical expenses, a key element in China’s 2009 health reforms implemented financial risk protection against crippling medical expenses.^9^ Strategies such as expanding health insurance coverage, promoting insurance benefits packages, and exploring multiple insurance payment methods were key elements of the reform program.

 The healthcare delivery system in China is hospital-centered, which consists of three levels: primary healthcare institutions, secondary hospitals, and tertiary hospitals.^10^ Every four years, the government accreditation agency accredits the level of each hospital, based on a basket of indicators, including the size of hospitals (such as the number of beds) and the quantity and quality of medical equipment (such as X-ray machines and magnetic resonance imaging). For stroke treatment, primary hospitals are unable to provide timely treatment for many types of stroke, such as thrombolysis, but can provide essential drugs and daily care for some stroke patients in the rehabilitation period, such as Aspirin. Secondary and tertiary hospitals have the ability and qualification to build stroke centers, where stroke centers in secondary hospitals are required to be equipped with essential stroke-related departments, such as neurology, neurosurgery, and emergency medicine. Diagnosing and advanced treatment methods, such as *computerized tomography* scans, electrocardiogram, and thrombolysis, are also required.^11^ Stroke centers in tertiary hospitals are required to have more stroke-related departments than those in secondary hospitals, such as interventional medicine and neurological rehabilitation departments. Higher-level treating medical technologies are also required in tertiary hospitals, such as carotid endarterectomy and removal of intracranial hematoma, and stroke rehabilitation treatment must be a basic facility.^11^ Under the tiered diagnosis and treatment system, primary healthcare institutions were expected to play the role of gatekeeper, filtering patients for treatment in higher-level hospitals, and, second, provide rehabilitation for patients referred from high-level hospitals.^12^

 Treatment at China’s hierarchical medical system was patient determined, but economically constrained by different OOP expenses paid by the insurance schemes.^13^ Higher-level hospitals generally had lower reimbursement rates than lower-level hospitals, which meant patients with minor illnesses were encouraged to access primary health institutions or secondary hospitals before tertiary hospitals.

 The financial protection role played by health insurance was to pay the non-OOP expenses directly to the health providers. Insurance payment methods shaped whether, what type, and how much healthcare was provided^14^, such as average length of hospital stay (ALOS)^15^ and scope of hospital treatments.^16^ After the 2009 reforms, four payment methods were implemented: fee-for-service (FFS), global budget (GB), bundled payment, and capitation payment. Each payment method was designed by the Medical Insurance Bureau (MIB), the government’s health insurance fund management agency, which meant the MIB formulated the OOP payment level for patients and the payment packages to hospitals. For each hospital, the local MIB managed the payment system separately for outpatient and inpatient treatments and for the two urban insurance schemes, the Urban Employee Basic Medical Insurance (UEBMI) and the Urban Residents Basic Medical Insurance (URBMI). Since different hospital levels played different stroke treatment roles, we hypothesize that different payment methods would suit different hospital levels.

Table 1 sets out the key characteristics of the four different payment methods.^14^ Using the FFS method, the MIB compensated the insurance providers according to an agreed price schedule and the amount of health services hospitals provided to patients. Consequently, hospitals were incentivized to overprescribe treatment, especially drugs and diagnostic tests, which resulted in rapidly increasing medical costs.^17^ GB, bundled payments, and capitation payments belonged to the prospective payment system. Under the prospective payment system, hospitals were paid in advance for treatments and the hospitals were responsible for costs in excess of any prospective payment, but retained any unspent funds as a profit.^18^ GB involved a prepaid lumpsum for treating patients within an agreed period, which limited the risk of overpayments for excessive or unnecessary treatments.^18^ The amount of health expenditure prepaid by the MIB for different hospitals was based on various factors, such as medical service revenue in the former three years, amount of unreasonable medical expenditure and the scale of the hospital.^19^ GB reimbursed patients a share of total hospital costs prepaid by MIB to the hospital, with all remaining hospital costs paid as OOP expenses by insured patients. Under the bundled payment system, once patients were diagnosed with a disease in the List of Diseases Covered by Insurance, they were expected to pay a fixed OOP amount for their entire treatment, with all remaining hospital treatment costs (such as diagnosis, medical examinations and treatment services) funded by the insurance fund, which was prepaid by the MIB. Diseases in the List of Diseases Covered by Insurance were usually selected according to the disease spectrum and health service capacity of health providers, with the List varying across different hospitals. Bundled payment treatment was conducted according to a reasonable standard of clinical pathway to ensure quality of health services. Under the bundled package, the amount of prepaid expenses to hospitals was based on the predicted number of cases and payment standard. In terms of the capitation payments, insurance members choose several, but a limited number of hospitals, which generally included one primary hospital, with which to sign contracts for health services. MIB prepaid the contracted hospital selected by the insured member. The contracted hospital had total responsibility for the contracted patient’s treatment, offering a set of medical services for a fixed period. Patients must seek health services in their choice of contracted hospitals. The amount of prepaid expense for hospitals was based on the number of patients contracted and payment standard of patients, which varied by insurance type and was decided by the MIB.

**Table 1:** Four Basic Payment Methods

**Payment Method**	**Unit of Payment**	**Common Term**	**Form of Payment**	**Comment**
1. Fee-for-service	Per service	FFS	Post-payment	Separate payments are often made for multiple services per day.
2. Bundled payment	Per episode	Payment per stay, case rates, quota payment, and case-based payment	Prospective	Covering related clinical services for an entire episode of care.
3. GB	Per time period	Budget-based payment	Prospective	MIB typically pays a certain cost to health services providers for an agreed time.
4. Capitation payment	Per beneficiary	Capitation	Prospective	Hospitals chosen by patient as contract hospitals will be prepaid by MIB.

Abbreviations: MIB, Medical Insurance Bureau; GB, global budget.

 Under the prospective payment system, MIB paid an amount of money to hospitals, where the hospital had total flexibility on how the treatment was provided. Considering the potential financial risk to patients, prospective payments to hospitals discouraged hospitals’ oversupplying medical services, motivated hospitals to contain medical expenses and contained patient OOP expenses.^18^ In 2017, the Chinese government proposed that the payment methods should be adjusted according to the type of illness.^18^ For example, the capitation payment method was applied to chronic diseases, while patients with complicated diseases that did not fit bundled payments, were directed to FFS. Stroke is considered both a chronic and critical illness, but the preferred payment method has not been yet determined. This is surprising since China has the highest incidence of stroke globally,^18^ with stroke costing about $US5.9 billion to China’s healthcare system annually in 2010.^18^ Given the high incidence of stroke, assessing which payment method provides the lowest total hospital costs for the medical system and lowest OOP expenses for stroke patients is an urgent priority for patients, families, and the health system.

 Recent studies have compared the effect of different payment methods on patients’ health expenditure. Using data from the 2013 National Health Services Utilization Survey, Yi et al^20^ found, GB, when compared to FFS, could significantly reduce total medical expenses and OOP expenses for hypertension patients. When assessing the effect of bundled payments on reducing cesarean delivery costs, Meng et al^21^ found that after replacing the FFS with the bundled payment method, OOP payments decreased significantly. An empirical study conducted by Jing et al^22^ reported that using the capitation payment method, total medical expenditures of the insured rural population decreased by 3.6%. In general, previous studies indicated that different payment methods have different effects on health service expenses. However, most of these studies have been limited to assessing the policy effect of a single new payment method compared with traditional payment methods.

 The present study aims to identify whether there are differences in urban stroke inpatients and outpatients’ OOP expenses and total hospital costs among the four payment methods for different hospital levels. We clarify which payment method resulted in the lowest OOP expenses for patients and the lowest hospital costs for the health system. From the cost perspective, the paper explores the most suitable payment method for different hospital levels for urban stroke patients.

## Methods

###  Data Source

 Data used in the present study comprise a 5% random sample of the UEBMI and URBMI insurance claims, which was sampled by the China Health Insurance Research Association (CHIRA) using a systematic random sampling strategy. The database constructed by CHIRA includes more than 80 main cities with well-established health insurance information systems in China. The previous study has demonstrated the sampling process in detail.^23^ In brief, with a random start, each Kth record from a population of size N was selected within the local health insurance database. With such a method, a sample size of n was obtained in each city, where N/n > =K. From the year 2013 to 2016, our data contains medical information on each stroke patient, including demographic information, expenditures of outpatient and inpatients services, and the payment method. The final sample included 400 672 stroke patients, based on the 10th revision of the International Statistical Classification of Diseases (ICD-10) codes (G45.8, G45.9, I60.1, I61.0-6, I61.9, I63.0-6, I63.8-9, I64.X).

###  Measures and Variables

 We conducted a cross-sectional study to estimate the differences in stroke health expenditure between the four payment methods. The main outcome variables were total hospital costs, which is the true measured cost of services, and OOP expense per visit, or the share of hospital costs paid by patients per visit. The reimbursement rate, or the percentage of total hospital costs paid to the hospitals determined by MIB, measured the actual financial protection provided by insurance and the inpatient ALOS reflected healthcare utilization by stroke inpatients. The primary independent variables were the payment method (FFS, GB, bundled, and capitation) and the level of hospital (primary, secondary, and tertiary hospitals). Outpatient and inpatient treatments are funded differently by MIB under the four payment methods, therefore, the inpatient-outpatient categories were also important independent variables. CHIRA only provided data on the hospital’s primary payment method, which did not allow us to identify any hospitals with mixed payment methods. The control variables included sex (male and female), age group (0-44, 45-64, 65-74, 75-79, 80 years or older), insurance type (UEBMI and URBMI), the Charlson Comorbidity Index (CCI) of patients,^24^ and 7 regions (South China—Guangdong, Guangxi, Hainan; East China—Shandong, Jiangsu, Shanghai, Zhejiang, Anhui, Jiangxi, Fujian; Southwest—Sichuan, Yunnan, Guizhou, Chongqing, Xizang; North China—Hebei, Beijing, Tianjin, Shanxi, Inner Mongolia; Northwest—Xinjiang, Ningxia, Qinghai, Shaanxi, Gansu; Central China—Henan, Hubei, Hunan and Northeast—Heilongjiang, Jilin, Liaoning) and year (2013, 2014, 2015, 2016). The average annual US$-RMB exchange rate from 2013 to 2016, US$0.159, was used to convert RMB into US dollars.

###  Statistical Analysis

 Descriptive analysis was used to analyze the demographic information and hospital costs, with the Kruskal–Wallis test used to evaluate the differences in total hospital cost, OOP expenses, and reimbursement rate by different payment methods. The generalized linear model (GLM) with a gamma distribution and a log link was used to explore the association of the payment method with medical costs by hospital level. Gender, age, insurance type, CCI, region, and year played the role of covariable in each GLM model. Statistical analyses were conducted using STATA version 14.0 (Stata Corp LP, College Station, TX) and a *P* value of less than.05 was considered statistically significant.

## Results

###  Patient Characteristics, Hospital Level and Payment Method

 As shown in Table 2, among the 406 224 stroke patients, 286 555 (70.5%) were outpatients, with an average age of 66.7, and 119 669 (29.5%) were inpatients, with an average age of 68.2. Male patients accounted for 58.2%, and UEBMI patients accounted for 89.4%, of the sample. Among outpatients, 34.0% were treated in primary hospitals, 22.5% in secondary hospitals and 43.4% in tertiary hospitals, and 43.4% of inpatients were treated in tertiary hospitals. The dominant payment method for outpatient healthcare was FFS (80.9%), while GB (44.9%) was the most used payment method for inpatient healthcare. More than half of patients had no comorbidities (55.9%) and North China accounted for 74.2% of outpatients and 24.5% of inpatient stroke patients (24.5%) in the sample. Patients in 2013 accounted for the largest proportion (30.7%) of the sample, with the number of patients in 2014-2016 roughly accounting for one-quarter of the sample.

**Table 2:** Socio-demographic Characteristics of Stroke Patients 2013 to 2016

**Characteristic**s	**Overall**	**Outpatient**	**Inpatient**
Gender, No. (%)			
Female	167 364 (41.8)	118 818 (42.1)	48 546 (41.1)
Male	233 308 (58.2)	163740 (57.9)	69 568 (58.9)
Age (y)			
Mean ± SD	67.1 ± 11.8	66.7 ± 11.8	68.2 ± 11.7
Insurance type, No. (%)			
UEBMI	358 023 (89.4)	265 750 (94.1)	92 273 (78.1)
URBMI	42 649 (10.6)	16 808 (5.9)	25 841 (21.9)
Hospital level, No. (%)			
Primary	114 916 (28.7)	96 210 (34.0)	18 706 (15.8)
Secondary	102 403 (25.6)	63 702 (22.5)	38 701 (32.8)
Tertiary	183 353 (45.8)	122 646 (43.4)	60 707 (51.4)
Payment methods, No. (%)			
FFS	280 364 (70.0)	228 503 (80.9)	51 861 (43.9)
GB	93 608 (23.4)	40 601 (14.4)	53 007 (44.9)
Bundled payment	1864 (0.5)	0 (0.0)	1864 (1.6)
Capitation payment	24 836 (6.2)	13 454 (4.8)	11 382 (9.6)
CCI, No. (%)			
0	358 789 (89.6)	251 979 (89.2)	106 810 (90.4)
1	37 728 (9.4)	29 263 (10.4)	8465 (7.2)
≥2	4155 (1.0)	1316 (0.5)	2839 (2.4)
Region, No. (%)			
South China	46 663 (11.6)	40 187 (14.2)	6476 (5.5)
East China	47 187 (11.8)	26 037 (9.2)	21 150 (17.9)
Southwest	20 152 (5.0)	4061 (1.4)	16 091 (13.6)
North China	238 485 (59.5)	209 521 (74.2)	28 964 (24.5)
Northwest	8172 (2.0)	677 (0.2)	7495 (6.3)
Central China	18 109 (4.5)	672 (0.2)	17 437 (14.8)
Northeast	21 904 (5.5)	1403 (0.5)	20 501 (17.4)
Year, No. (%)			
2013	123 148 (30.7)	86 785 (30.7)	36 363 (30.8)
2014	96 973 (24.2)	72 193 (25.6)	24 780 (21.0)
2015	86 735 (21.7)	63 694 (22.5)	23 041 (19.5)
2016	93 816 (23.4)	59 886 (21.2)	33 930 (28.7)
Total	400 672	282 558 (70.5)	118 114 (29.5)

Abbreviations: SD, standard deviation; UEBMI, Urban Employee Basic Medical Insurance; URBMI, Urban Resident Basic Medical Insurance; CCI, Charlson Comorbidity Index; FFS, fee-for-service; GB, global budget.

###  Outpatient Hospital and Out-of-Pocket Expenditure by Payment Method and Hospital Level

Table 3 presents the outpatient expenses for stroke care by payment method and hospital level. In primary hospitals, the capitation payment system had the lowest total hospital costs (RMB180.9/US$28.8) and the lowest OOP expenses (RMB75.6/ US$12.0) per visit. FFS had the highest reimbursement rate, 78.4%, but the highest total hospital costs (RMB371.3/US$59.0) per visit, while under the GB system, patients faced the highest OOP expenses (RMB97/US$15.4). In secondary hospitals, FFS had the highest reimbursement rate, 75.1%, and lowest OOP expenses (RMB123.4/US$19.6), but the highest total hospital costs (RMB495.8/US$78.8) per visit. GB had the lowest total hospital costs (RMB344.7/US$54.8), while the capitation payment had the lowest reimbursement rate, only 35.0%, and the highest OOP expenses (RMB308.6/US$49.1) per visit. In tertiary hospitals, the capitation payment saw patients face the highest total hospital costs (RMB635.7/US$101.1) per visit, while the FFS had the lowest OOP expense per visit, RMB151.8/US$24.1. GB payment had the lowest total hospital costs (RMB495.3/US$78.8), but the highest OOP expenses (RMB249.8/US$39.7).

**Table 3:** Outpatient Costs by Payment Method and Hospital Level

	**FFS**	**GB**	**Capitation Paymen**t	***P*** ** Value**
**Primary**				
Total hospital costs (RMB)	371.3	222.0	180.9	<.001
SD	403.7	214.0	209.2	
OOP expenses (RMB)	80.1	97.0	75.6	<.001
SD	139.0	143.1	114.7	
Reimbursement rate	78.4%	56.3%	58.2%	<.001
**Secondary**				
Total hospital costs (RMB)	495.8	344.7	475.1	<.001
SD	521.9	300.3	435.2	
OOP expenses (RMB)	123.4	167.2	308.6	<.001
SD	189.3	197.8	310.3	
Reimbursement rate	75.1%	51.5%	35.0%	<.001
**Tertiary**				
Total hospital costs (RMB)	579.5	495.3	635.7	<.001
SD	607.5	479.0	756.8	
OOP expenses (RMB)	151.8	294.8	152.2	<.001
SD	237.2	344.9	293.5	
Reimbursement rate	73.8%	40.5%	76.1%	<.001
**Average**				
Total hospital costs (RMB)	492.4	375.3	310.6	<.001
SD	536.8	392.9	398.5	
OOP expenses (RMB)	121.9	203.7	156.2	<.001
SD	201.4	278.6	238.3	
Reimbursement rate	75.2%	45.7%	49.7%	<.001

Abbreviations: SD, standard deviation; OOP, out-of-pocket; FFS, fee-for-service; GB, global budget.

###  Inpatient Hospital Expenditures, OOP Expenditures and ALOS by Payment Method and Hospital Level

Table 4 presents the inpatient expenses for stroke care by payment method and hospital level. In primary hospitals, patients had the lowest total hospital cost (RMB5918.7/US$941.1) per visit under FFS, while patients under the capitation payment had the lowest OOP expenses (RMB876.5/US$139.4) and the longest ALOS (14.9 days) per visit. In secondary hospitals, the capitation payment system incurred the lowest total hospital costs (RMB7342.9/US$1167.5) and OOP expenses (RMB1664.2/US$185.1), and the second-lowest ALOS (12.2 days). FFS accounted for the highest total hospital cost (RMB10 838.7/US$1723.4) and OOP expenses (RMB2756.7/US$438.3) and the longest ALOS (14.1 days) in secondary hospitals. In tertiary hospitals, the capitation payment system had the highest reimbursement rate, 81.5%, the lowest total hospital costs (RMB17 711.7/US$2816.2), and OOP expenses (RMB3276.3/US$520.9) and second-lowest ALOS (15.2 days). For inpatients, the FFS payment method had the longest ALOS (16.4 days).

**Table 4:** Inpatient Costs and ALOS by Payment Method and Hospital Level

	**FFS**	**GB**	**Bundled Payment**	**Capitation Payment**	***P*** ** Value**
**Primary**					
Total hospital costs (RMB)	5918.7	6266.7		7190.3	<.001
SD	13 460.9	13 974.7		12 023.3	
OOP expenses (RMB)	1342.6	1764.7		876.5	<.001
SD	6335.5	6769.0		2075.5	
Reimbursement rate	79.0%	71.8%		87.8%	<.001
ALOS	11.7	12.0		14.9	<.001
**Secondary**					
Total hospital costs (RMB)	10 838.7	8877.8	8155.6	7342.9	<.001
SD	26 307.0	11 524.3	11 158.8	9196.3	
OOP expenses (RMB)	2756.7	2043.0	2290.0	1664.2	<.001
SD	16 740.6	3946.3	5334.3	3741.1	
Reimbursement rate	74.6%	77.0%	71.9%	77.3%	<.001
ALOS	14.1	13.1	11.6	12.2	<.001
**Tertiary**					
Total hospital costs (RMB)	19 529.3	19 003.1	19 155.4	17 711.7	<.001
SD	35 619.2	26 799.5	25 901.0	27 291.2	
OOP expenses (RMB)	5343.8	5677.9	5077.3	3276.3	<.001
SD	18 571.1	12 385.1	8214.1	6752.2	
Reimbursement rate	72.6%	70.1%	73.5%	81.5%	<.001
ALOS	16.4	15.4	15.1	15.2	<.001
**Average**					
Total hospital costs (RMB)	13 860.7	13 674.5	12 363.2	15 619.0	<.001
SD	29 788.2	21 791.3	19022.3	25 129.0	
OOP expenses (RMB)	3665.9	3869.6	3356.2	2922.9	<.001
SD	16 327.4	9735.6	6722.1	6276.0	
Average reimbursement rate	73.6%	71.7%	72.9%	81.3%	<.001
ALOS	14.7	14.1	12.1	14.7	<.001

Abbreviations: SD, standard deviation; OOP, out-of-pocket; FFS, fee-for-service; GB, global budget; ALOS, average length of hospital stay.

###  Association Between Payment Method and Expenditure by Hospital Level 

Table 5 displays the association between the payment method and expenditures by hospital level. In primary hospitals, the total outpatient hospital costs under the capitation payment system was 49.2% (=exp ^-0.745^–1) (95% CI = -0.50, -0.48), and OOP expenses 1.2% (95% CI = -0.02, -0.005), lower than that under the FFS. In secondary hospitals, inpatients covered by the GB (-18.6%, 95% CI = -0.22, -0.16), bundled payment (-25.2%, 95% CI = -0.33, -0.17), and capitation payment (-34.9%, 95% CI = -0.40, -0.29) had significantly lower total hospital costs than those covered by the FFS system. However, the GB payment system incurred 37.9% (95% CI = 0.34, 0.43) and the capitation payment incurred 166.5% (95% CI = 1.55, 1.79) higher OOP expenses than the FFS for outpatients. In tertiary hospitals, the capitation payment system had much lower total hospital costs (-9.2%, 95% CI = -0.13, -0.06) and OOP expenses (-33.4%, 95% CI = -0.38, -0.29) for inpatients than the FFS system. Under the GB system, patients faced 5.2% (CI = 0.001, 0.11) higher OOP expenses than under the FFS.

**Table 5:** Payment Method and Health Costs by Hospital Level: Coefficient and 95% CI

**Total Hospital Costs**	**Primary Hospital**		**Secondary Hospital**		**Tertiary Hospital**	
	**Outpatient **	**Inpatient**	**Outpatient **	**Inpatient **	**Outpatient **	**Inpatient**
Payment method (Ref: FFS)						
GB	-0.33^a^ (-0.34, -0.31)	-0.02 (-0.08, 0.04)	-0.27^a^ (-0.32, -0.29)	-0.19^a^ (-0.22, -0.16)	-0.12^a^ (-0.14, -0.11)	-0.046^a^ (-0.07, -0.02)
Bundled payment	-	-	-	-0.25^a^ (-0.33, -0.17)	-	-0.04 (-0.15, 0.08)
Capitation payment	-0.49^a^ (-0.50, -0.48)	0.10 (-0.09, 0.33)	-0.003 (-0.03, 0.03)	-0.35^a^ (-0.40, -0.29)	0.23^a^ (0.15, 0.31)	-0.09^a^ (-0.13, -0.06)
**OOP Expenses**						
Payment method (Ref: FFS)						
GB	0.20^a^ (0.16, 0.24)	-0.001 (-0.11, 0.12)	0.38^a^ (0.34, 0.43)	-0.24^a^ (-0.30, -0.18)	0.94^a^ (0.90, 0.99)	0.052^b^ (0.001, 0.11)
Bundled payment	-	-	-	-0.08 (-0.27, 0.15)	-	-0.13 (-0.30, 0.09)
Capitation payment	-0.01^b^ (-0.02, -0.005)	-0.40^a^ (-0.58, -0.15)	1.66^a^ (1.55, 1.79)	-0.40^a^ (-0.50, -0.28)	0.053 (-0.04, 0.16)	-0.33^a^ (-0.38, -0.29)

Abbreviations: OOP, out-of-pocket; FFS, fee-for-service; GB, global budget; CI, confidence interval. All models were adjusted for gender, age, insurance type, CCI, region, and year. All the regression results (β) have been transformed using the formula, *Coefficient = e*^β^*– 1.* ^a^ *P* <.001, ^b^*P* <.05.

## Discussion

 Based on the claim database of urban insurance schemes covering over 95% of the urban insured in China, the present study provides a comprehensive view of differences in hospital costs and OOP expenses for patients with stroke under different payment methods and different hospital levels. There were significant differences in the hospital’s total costs and patients’ OOP expenses for healthcare. The capitation payment system displayed the lowest total hospital costs and lowest OOP expenses for outpatients in primary hospitals and inpatients in secondary and tertiary hospitals. The capitation payment system is widely considered a reasonable payment method for patients in primary hospitals.^25-27^ Disease prevention, illness treatment, rehabilitation care, medical referral services, and health education were all important functions for primary hospitals, and capitation payment promoted these functions. Since hospitals are prepaid fixed healthcare amounts or reimbursements, the best way to maximize their profit was to provide health services at the lowest total cost. Primary hospitals took four main measures to reduce total costs: prevention to keep contracted patients healthy and reduce the incidence of illness by providing health education^28^; prevent or identify early catastrophic illnesses through regular check-ups and by educating patients to seek early treatment for illnesses; prompt referral of patients with serious illness to higher-level hospitals; and rehabilitation care for patients referred back from higher-level hospitals. These measures corresponded with the main functions of primary hospitals. For primary hospital inpatients, the results in Table 4 indicated that inpatients covered by the capitation payment system had the lowest OOP expenses, but the longest long ALOS due to high levels of rehabilitation care, compared to inpatients using other payment methods in primary hospitals. Capitation payment also resulted in lower OOP expenses for stroke inpatients and lower total hospital costs for secondary and tertiary hospitals. This is consistent with Ponce and colleagues’^29^ finding that patients under capitation payment system had 29% lower costs on pharmaceuticals and 21% lower costs on laboratory services than those using FFS in Portugal. We speculate that under the capitation payment hospitals competed with other hospitals to enlist and retain contracted patients to get the capitation reimbursement. Various cost-saving measures, such as the standardization of care and more effective therapeutic schedules, were implemented by contracted hospitals to reduce patients’ OOP expenses, hospital total costs, win patient trust, and maximize the hospital’s income. Importantly, Chen et al^30^ found that the capitation payment significantly decreased the OOP expense of patients without compromising the quality of healthcare.

 Our results show that the GB system had lower total hospital costs than FFS for outpatients in primary, secondary, and tertiary hospitals, with FFS patients incurring higher OOP expenses. Similarly, Han et al^31^ found that outpatients under the GB system had 23.15% lower total expense per visit than those covered by the traditional FFS system. As a prospective payment system, the GB subjects the hospitals to economic risk since the budget is always prepaid to a single hospital, and the hospital will be responsible for its own profit and loss. Consequently, GB incentivizes hospitals to engage in cost-saving treatments.^32,33^ The results in Table 3 that show the GB reimbursement rate was significantly lower than FFS support this argument.

 The aim of the payment method reform was to change hospital treatment behavior through offering patient healthcare choice.^34^ From the cost perspective, when a payment method had both the lowest total hospital costs to the medical system and the lowest OOP expenses for insured patients, we posit that the payment method was the “best payment method.” Only the capitation payment displayed both the lowest total hospital costs and lowest OOP expenses for outpatients in primary hospitals and inpatients in secondary and tertiary hospitals. Under capitation payment, hospitals have the incentive to minimize hospital costs to retain larger care of the prepaid fund, contain OOP expenses to reduce patients’ economic burden, and win patient trust. Recent research has shown that under capitation payments, patients with various types of illnesses received equal quality of healthcare compared with previous payment methods,^30,35-37^ which suggests that the capitation payment method should be implemented for primary outpatient and secondary and tertiary inpatient care.

 For inpatients in primary hospitals, and outpatients in secondary and tertiary hospitals, the lowest OOP expense and lowest total hospital costs payment method did not coincide. When the financial protection for patients and minimizing hospital costs do not coincide, the government health system needs to determine whether minimizing patient OOP expenses or minimizing hospital costs should be the main priority. Given the 2009 health reforms, payment reform, and the goal of “Healthy China 2030 Outline” aimed to alleviate patients’ financial health burden, especially reducing OOP payments, the government health policymakers have indicated that the lowest OOP expenses for patients were the primary aim of the payment method. The lowest OOP expense payment system was FFS for outpatient secondary hospitals; FFS or capitation for outpatient tertiary hospitals; and the capitation system for primary inpatients.

 The bundled payment system has been widely considered a superior policy tool on efficient terms, controlling health expenditure and improving the performance of the healthcare system.^38^ Similar to other studies, we found both hospital costs and OOP expenses of patients under bundled payment were lower than those under the previous FFS system in secondary and tertiary hospitals.^39,40^ Also, the ALOS generally decreased under the bundled payment, with lower readmission rates for patients as well.^40^ Since stroke is generally characterized by a well-defined episode stage for care, stroke is considered a suitable candidate for bundled payment.^41^ Surprisingly, the bundled payment did not display either the lowest hospital costs or OOP expenses in secondary and tertiary hospitals. Previous studies have revealed large variation in cost for patients with stroke across hospitals due to inefficient care in the clinical process.^41,42^ Improving the quality of treatment and standardizing the clinical process to improve the efficiency of stroke care may be required to promote bundled payment for stroke.

###  Limitations

 This study has several limitations. First, we are unable to assess the quality of healthcare under different payment methods due to a lack of related clinical information on each patient. All our policy suggestions were from the perspective of cost, which is consistent with results from previous studies on the “best payment method” for different hospital levels in China. Second, we could not differentiate mixed payment methods in some hospitals as we have only the primary payment method provided by the CHIRA data. Third, as stroke patients were identified based on their ICD-10 illness code, we could not differentiate whether the cases are stroke episodes or individual patients. Finally, extrapolation from our findings to the stroke patients under the rural health insurance scheme requires caution since our claims database only provided evidence on the urban population. Whether our stoke findings apply to other illnesses also requires further study.

## Conclusion

 The choice of payment system has important implications for patients’ OOP expenses, the financial well-being of Chinese families, the hospital systems’ operational sustainability, and the viability of Chinese healthcare. Total hospital costs and OOP expenses of stroke patients varied by different payment methods and different hospital levels. For the health system and patient OOP expenses, the capitation payment was performed as the most economical payment method, offering both the lowest total hospital costs and the lowest OOP expenses for outpatients in primary hospitals and inpatients in secondary hospitals and tertiary hospitals. When the lowest OOP expenses and lowest total hospital costs do not coincide, there was no mutual “best payment method” for the hospital system and patients, which requires further studies of payment method and treatment quality. For primary inpatients and outpatients in secondary and tertiary hospitals, a mixed payment system must be applied. Healthcare policymakers need to clearly state whether minimizing patient OOP expenses or minimizing hospital total costs should be the rule governing the payment method for stroke patients.

## Acknowledgements

 We thank the support of the China Health Insurance Research Association, for providing the valuable data.

## Ethical issues

 Since the claims data we used was an anonymized database and had no impact on patients’ health, the informed consent was exempted. This study was approved by the Ethics Committee of Beijing University of Chinese medicine (No.2019BZHYLL0201).

## Competing interests

 Authors declare that they have no competing interests.

## Authors’ contributions

 YY, XFS, and XWM designed this study and drafted the original manuscript. SN and EM developed the idea and participated in revising the paper; ZY and ZWH played an important role in analyzing the data and participated in drafting the manuscript; YM collected research data and critically revised the manuscript, all authors have read and approved the final manuscript.

## References

[1] Yang G, Wang Y, Zeng Y (2013). Rapid health transition in China, 1990-2010: findings from the Global Burden of Disease Study 2010. Lancet.

[2] Naghavi M, Abajobir AA, Abbafati C (2017). Global, regional, and national age-sex specific mortality for 264 causes of death, 1980-2016: a systematic analysis for the Global Burden of Disease Study 2016. Lancet.

[3] Jeong YG, Myong JP, Koo JW (2015). The modifying role of caregiver burden on predictors of quality of life of caregivers of hospitalized chronic stroke patients. Disabil Health J.

[4] Mei Y, Wilson S, Lin B, Li Y, Zhang Z (2018). Benefit finding for Chinese family caregivers of community-dwelling stroke survivors: a cross-sectional study. J Clin Nurs.

[5] Caro CC, Costa JD, Da Cruz DMC (2018). Burden and quality of life of family caregivers of stroke patients. OccupTher Health Care.

[6] Xu K, Evans DB, Kawabata K, Zeramdini R, Klavus J, Murray CJ (2003). Household catastrophic health expenditure: a multicountry analysis. Lancet.

[7] Li Y, Wu Q, Xu L (2012). Factors affecting catastrophic health expenditure and impoverishment from medical expenses in China: policy implications of universal health insurance. Bull World Health Organ.

[8] Wang Q, Liu H, Lu ZX, Luo Q, Liu JA (2014). Role of the new rural cooperative medical system in alleviating catastrophic medical payments for hypertension, stroke and coronary heart disease in poor rural areas of China. BMC Public Health.

[9] Yip W, Fu H, Chen AT (2019). 10 years of health-care reform in China: progress and gaps in universal health coverage. Lancet.

[10] Zhou Z, Zhao Y, Shen C, Lai S, Nawaz R, Gao J (2021). Evaluating the effect of hierarchical medical system on health seeking behavior: a difference-in-differences analysis in China. Soc Sci Med.

[11] General Office of the National Health and Family Planning Commission. Guiding Principles for the Construction and Management of Hospital Stroke Centers. 2016. http://www.nhc.gov.cn/yzygj/s3593/201611/efb995886bfe423a84d4a4760ee4a67f.shtml. Accessed June 26, 2021.

[12] Herong G (2017). Health governance and system of tiered diagnosis and treatment in China. Journal of Public Management.

[13] Shen M, He W, Li L (2020). Incentives to use primary care and their impact on healthcare utilization: evidence using a public health insurance dataset in China. Soc Sci Med.

[14] Quinn K (2015). The 8 basic payment methods in health care. Ann Intern Med.

[15] Coulam RF, Gaumer GL (1992). Medicare’s prospective payment system: a critical appraisal. Health Care Financ Rev.

[16] Hervis RM (1993). Impact of DRGs on the medical profession. Clin Lab Sci.

[17] Yip WC, Hsiao W, Meng Q, Chen W, Sun X (2010). Realignment of incentives for health-care providers in China. Lancet.

[18] Li HM, Chen YC, Gao HX (2018). Effectiveness evaluation of quota payment for specific diseases under global budget: a typical provider payment system reform in rural China. BMC Health Serv Res.

[19] Lu Y, Tao H, Huang Y, Wang Y, Liu Y, Zhang T (2021). Problems and countermeasures of medical insurance fund allocation under global budget system. Health Econ Res.

[20] Huang Y, Liu Y, Yang X, Li J, Fang P (2016). Global budget payment system helps to reduce outpatient medical expenditure of hypertension in China. Springerplus.

[21] Meng Z, Zou K, Ding N, Zhu M, Cai Y, Wu H (2019). Cesarean delivery rates, costs and readmission of childbirth in the new cooperative medical scheme after implementation of an episode-based bundled payment (EBP) policy. BMC Public Health.

[22] Jing L, Bai J, Sun X (2016). NRCMS capitation reform and effect evaluation in Pudong New Area of Shanghai. Int J Health Plann Manage.

[23] Yong M. Research on Medical Expenses and Influencing Factors of Stroke Patients in Chinese Urban Residents. Beijing University of Chinese; 2018.

[24] Quan H, Li B, Couris CM (2011). Updating and validating the Charlson comorbidity index and score for risk adjustment in hospital discharge abstracts using data from 6 countries. Am J Epidemiol.

[25] General Office of the State Council. Guiding Opinions of the General Office of the State Council on Further Deepening the Reform of Basic Medical Insurance Payment Methods. 2017. http://www.gov.cn/zhengce/content/2017-06/28/content_5206315.htm. Accessed April 26, 2020.

[26] Andoh-Adjei FX, Spaan E, Asante FA, Mensah SA, van der Velden K (2016). A narrative synthesis of illustrative evidence on effects of capitation payment for primary care: lessons for Ghana and other low/middle-income countries. Ghana Med J.

[27] Yun Z, Xiao-yan P (2013). Research on strengthening health management function of basic medical institutions by capitation payment. Chinese Health Economics.

[28] Pearson WS, King DE, Richards C (2013). Capitated payments to primary care providers and the delivery of patient education. J Am Board Fam Med.

[29] Ponce P, Marcelli D, Guerreiro A (2012). Converting to a capitation system for dialysis payment--the Portuguese experience. Blood Purif.

[30] Gao C, Xu F, Liu GG (2014). Payment reform and changes in health care in China. Soc Sci Med.

[31] Zhang H, Zhang H, Ji B, Song N (2018). Comparative analysis of the average expense of outpatients in Beijing before and after the implementation of the global budget system. Chinese Health Quality Management.

[32] He R, Miao Y, Ye T (2017). The effects of global budget on cost control and readmission in rural China: a difference-in-difference analysis. J Med Econ.

[33] Ma CA, Mak HY (2015). Information disclosure and the equivalence of prospective payment and cost reimbursement. J Econ Behav Organ.

[34] Li H, Chen Y, Gao H (2019). Effect of an integrated payment system on the direct economic burden and readmission of rural cerebral infarction inpatients: evidence from Anhui, China. Int J Environ Res Public Health.

[35] Bamimore MA, Devlin RA, Zaric GS, Garg AX, Sarma S. Quality of diabetes care in blended fee-for-service and blended capitation payment systems. Can J Diabetes 2021;45(3):261-268.e211. 10.1016/j.jcjd.2020.09.002. 33162371

[36] Ukhanova M, Marino M, Angier H (2021). The impact of capitated payment on preventive care utilization in community health clinics. Prev Med.

[37] Ems D, Murty S, Loy B (2018). Alternative payment models in medical oncology: assessing quality-of-care outcomes under partial capitation. Am Health Drug Benefits.

[38] Jian W, Lu M, Chan KY (2015). Payment reform pilot in Beijing hospitals reduced expenditures and out-of-pocket payments per admission. Health Aff (Millwood).

[39] He R, Ye T, Wang J (2018). Medical service quality, efficiency and cost control effectiveness of upgraded case payment in rural China: a retrospective study. Int J Environ Res Public Health.

[40] Agarwal R, Liao JM, Gupta A, Navathe AS (2020). The impact of bundled payment on health care spending, utilization, and quality: a systematic review. Health Aff (Millwood).

[41] Matchar DB, Nguyen HV, Tian Y (2015). Bundled payment and care of acute stroke: what does it take to make it work?. Stroke.

[42] Miller DC, Gust C, Dimick JB, Birkmeyer N, Skinner J, Birkmeyer JD (2011). Large variations in Medicare payments for surgery highlight savings potential from bundled payment programs. Health Aff (Millwood).

